# Supplementary material for: Inactivation of GH3.5 by COP1-mediated K63-linked ubiquitination promotes seedling hypocotyl elongation
Source: Nat Commun. 2025 Apr 14;16:3541. doi: 10.1038/s41467-025-58767-6 (PMC11997217; doi:10.1038/s41467-025-58767-6)
Supplement: Supplementary file 1 — Supplementary Information [file 41467_2025_58767_MOESM1_ESM.pdf]

Supplementary Information

Inactivation of GH3.5 by COP1-mediated K63-linked ubiquitination promotes seedling hypocotyl elongation

Liu et al.

This document contains Supplementary Figs. 1 to 7, Supplementary Tables 1 and 2.

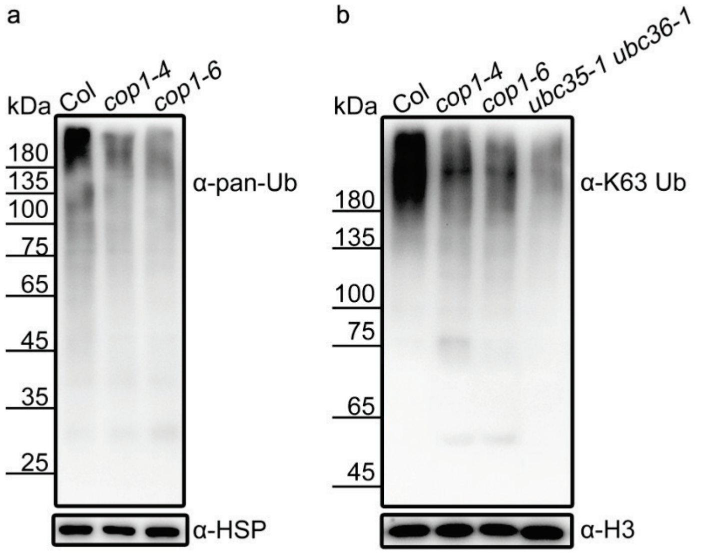

Supplementary Fig. 1 COP1 is involved in the formation of K63 polyubiquitin chains in the dark. a The total ubiquitination level in dark-grown Col and *cop1* mutants. α-pan-Ub, anti-pan-ubiquitin antibody; HSP was used as a loading control. **b** K63-linked ubiquitination levels in dark-grown Col, *cop1-4*, *cop1-6*, and *ubc35-1 ubc36-1*. α-K63 Ub, K63-linkage-specific polyubiquitin antibody. H3 was used as a loading control. Source data are provided as a Source Data file.

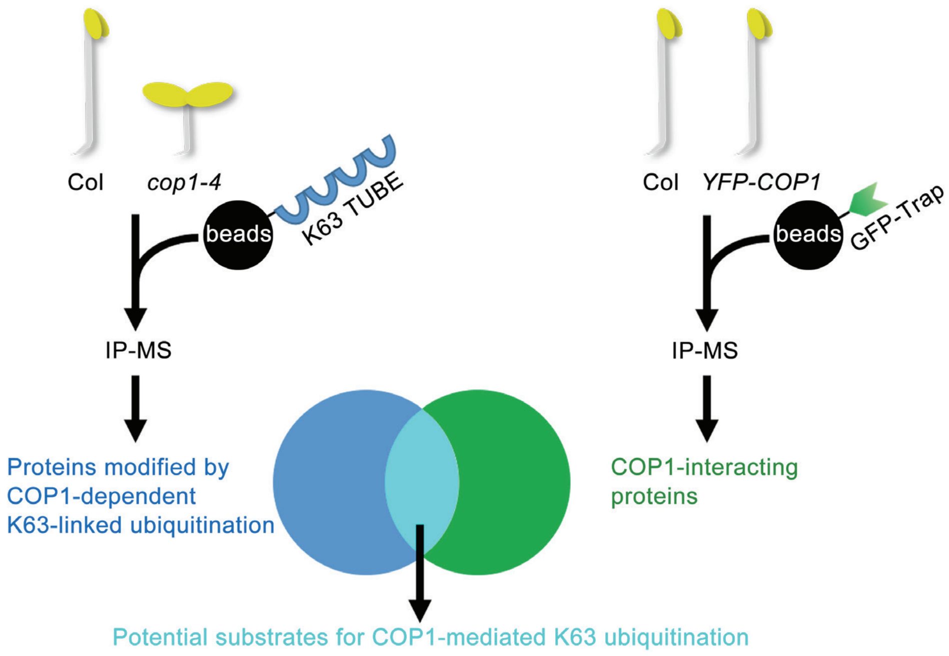

Supplementary Fig. 2 Scheme designed for screening of potential substrates for COP1-mediated K63 ubiquitination. Left panel, K63 TUBE-based IP-MS identified proteins whose K63 ubiquitination was dependent on COP1. Right panel, GFP-trap-based IP-MS identified COP1-interacting proteins.

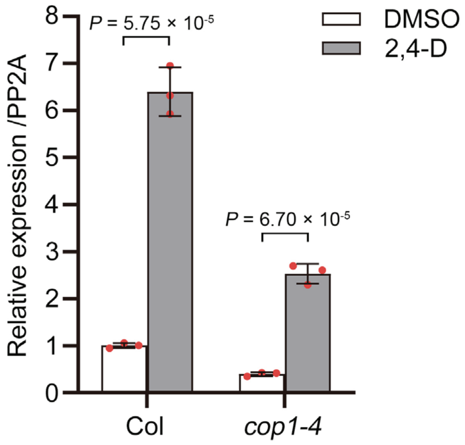

Supplementary Fig. 3 2,4-D treatment partially rescues *GH3.5* expression in *cop1-4* in the dark. Col and *cop1-4* seedlings were grown in the dark on MS medium supplemented with DMSO or 0.1 μM 2,4-D. Relative transcript levels were normalized to *PP2A*. Data are presented as the mean ± SD, $n = 3$ technical replicates. Statistically significant differences were determined by two-sided Student's *t*-test. RT-qPCR was independently repeated twice with similar results. Source data are provided as a Source Data file.

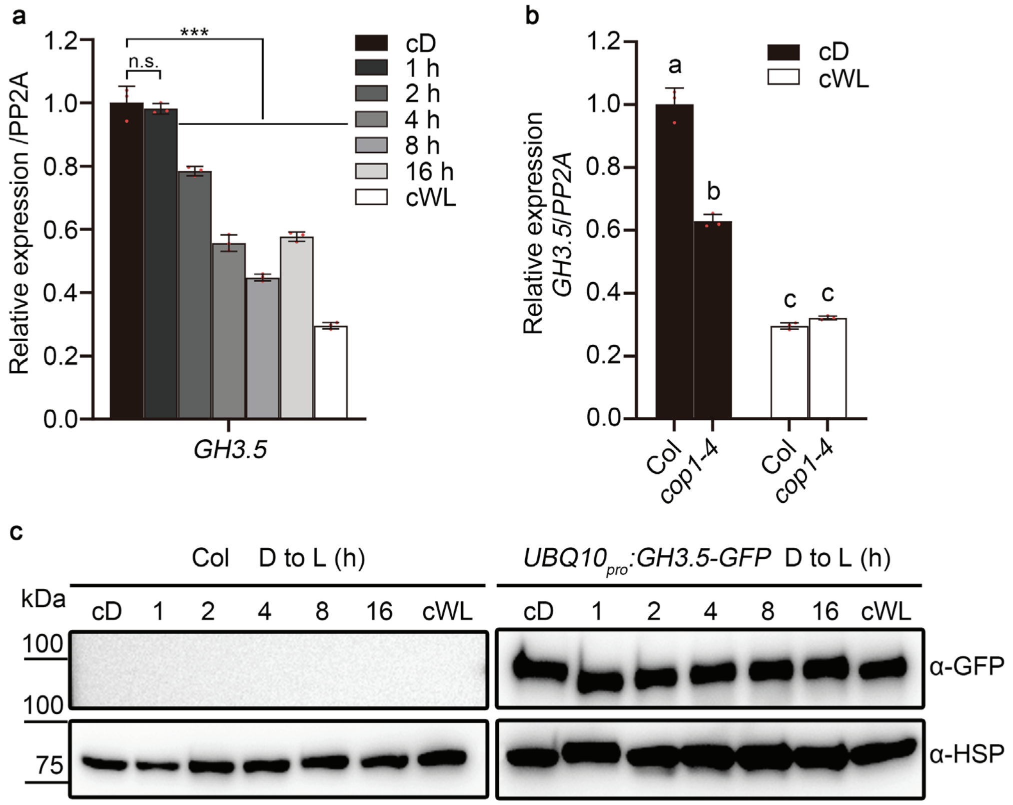

Supplementary Fig. 4 The effect of light irradiation on the regulation of mRNA and protein levels of GH3.5. a The relative *GH3.5* expression in Col responding to a switch from darkness to light. Relative transcript levels were normalized to *PP2A*. Data are presented as the mean ± SD, $n = 3$ technical replicates. cD, continuous darkness; cWL, continuous white light (55.07 μmol m^{-2} s^{-1}). Asterisks represent statistically significant differences (***$P < 0.001$) determined by ordinary one-way ANOVA with Dunnett's multiple comparisons test. n.s., not significant. **b** *GH3.5* expression levels in Col and *cop1-4* grown in the dark or light. Relative transcript levels were normalized to *PP2A*. Data are presented as the mean ± SD, $n = 3$ technical replicates. Different lowercase letters above the histogram represent statistically significant differences ($P < 0.001$) determined by ordinary two-way ANOVA with Tukey's post hoc test. The RT-qPCR experiments were independently repeated twice

with similar results. **c** Protein abundance of GH3.5 in the *GH3.5-GFP* overexpression line responding to a switch from dark to light. HSP was used as a loading control. Source data are provided as a Source Data file.

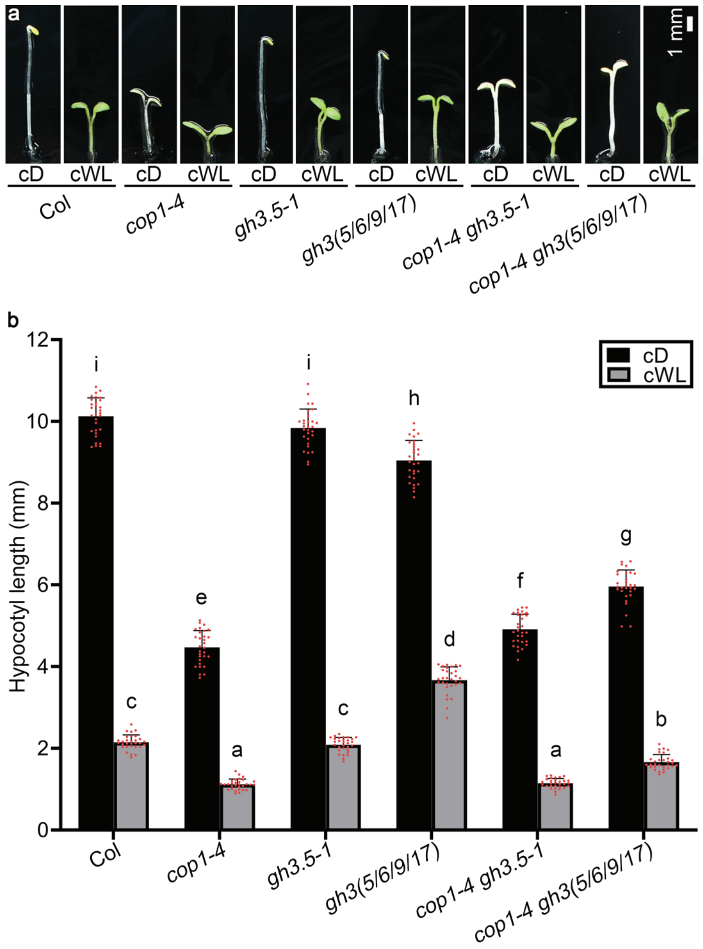

Supplementary Fig. 5 Light inhibits hypocotyl elongation by attenuating *COP1*-mediated inhibition of *GH3s* function. a Phenotypes of seedlings of Col, *cop1-4*, and *gh3* mutants in Col and *cop1-4* backgrounds. Seedlings were grown in continuous darkness (cD) or continuous white light (cWL, 35 μmol m^{-2} s^{-1}) for 4 days. Scale bar, 1 mm. **b** Hypocotyl lengths of seedlings as indicated in (**a**). Data are presented as the mean ± SD, n = 30 seedlings. Different lowercase letters above the histogram represent statistically significant differences ($P < 0.0001$) determined by ordinary two-way ANOVA with Tukey's post hoc test. Source data are provided as a Source Data file.

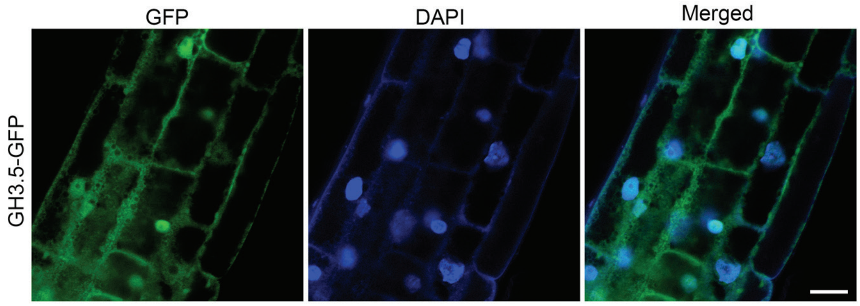

Supplementary Fig. 6 Subcellular localization of GH3.5 in vivo. *GH3.5-GFP* seedlings were grown in continuous darkness for 4 d. The fluorescence signals from the root cells were monitored. DAPI was used to label the nucleus. Scale bar, 10 μm. The experiment was independently repeated twice with similar results.

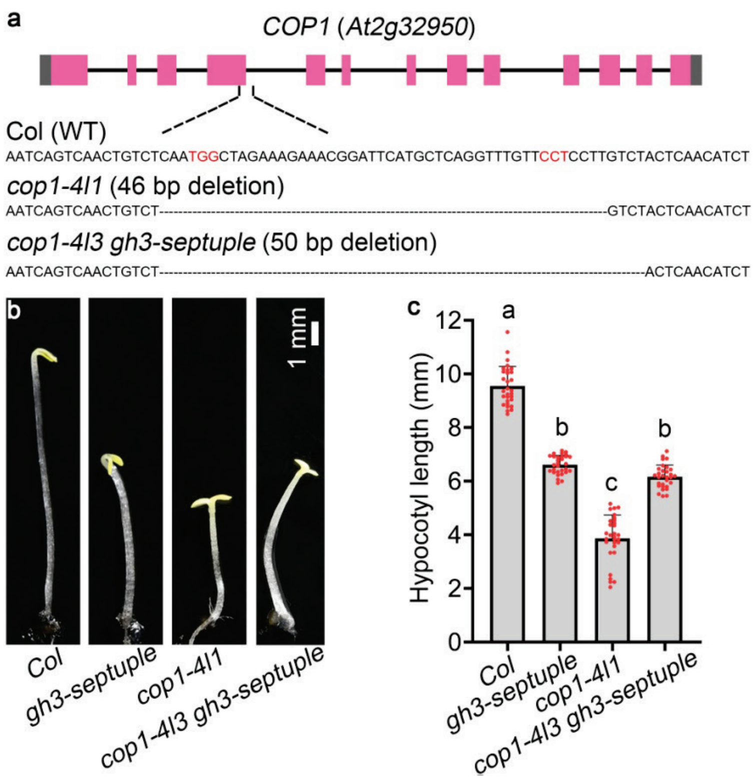

Supplementary Fig. 7 COP1 interacts genetically with the group II *GH3* gene family in the dark. a The *cop1-4 like* (*cop1-4l*) mutants generated in the background of Col and *gh3-septuple* by CRISPR/Cas9. Gray boxes in the gene body indicate the untranslated regions (UTR), pink boxes indicate the exon regions, and horizontal black lines indicate the intron regions. Red capital letters represent protospacer adjacent motif (PAM) sequences. Dashes, base deletions. *gh3-septuple*, *gh3(1/2/3/4/5/6/17)* septuple mutant. **b, c** Hypocotyl lengths of Col, *gh3-septuple*, *cop1-4l1*, and *cop1-4l3 gh3-septuple* seedlings grown in the dark for 4 days. Scale bar, 1 mm. Data are presented as the mean ± SD, n = 30 seedlings. Different lowercase letters above the histogram indicate statistically significant differences ($P < 0.0001$) determined by ordinary one-way ANOVA with Tukey's post hoc test. Source data are provided as a Source Data file.

Supplementary Table 1. The primer sequences used in the study.

Primer name	Primer sequence (5'-3')
**For Cloning**	
GH3.5-nLUC-F	ACGGGGGACGAGCTCGGTACCATGCCTGAGGCACCAAAG
GH3.5-nLUC-R	CGCGTACGAGATCTGGTCGACGTTACTCCCCCACTGTTTG
cLUC-COP1-F	TACGCGTCCCGGGGCGGTACCATGGAAGAGATTTCG
cLUC-COP1-R	ACGAAAGCTCTGCAGGTCGACTCACGCAGCGAGTACCAG
cLUC-COP1-N-R	ACGAAAGCTCTGCAGGTCGACTCACCGAAACTGATC
cLUC-COP1-C-F	TACGCGTCCCGGGGCGGTACCATGGAAGCACTACAAAGGGG
COP1-ΔRING-MF	GAGATTGGAGCACCGAATAATCAGCTTTAC
COP1-ΔRING-MR	GTAAAGCTGATTATTCGGTGCTCCAATCTC
COP1-ΔCoil-MF	TTGGATCAGTTTCGGAGGGACAGATATTCTG
COP1-ΔCoil-MR	CAGAATATCTGTCCCTCCGAAACTGATCCAA
COP1-ΔWD40-MF	CGTGATGATGAGCTGGAAATCACGAGACCC
COP1-ΔWD40-MR	GGGTCTCGTGATTTCCAGCTCATCATCACG
MBP-COP1-F	GAAGGATTTCAGAATTCATGGAAGAGATTTCG
MBP-COP1-R	CTTGCCTGCAGGTCGACTCACGCAGCGAGTACCAG
MBP-COP1-N-R	CTTGCCTGCAGGTCGACTCACCGAAACTGATC
MBP-COP1-C-F	GAAGGATTTCAGAATTCATGGAAGCACTACAAAGGGG
GST-GH3.5-F	CTGGTTCCGCGTGGATCCATGCCTGAGGCACCAAAG
GST-GH3.5-R	GAGTCGACCCGGGAATTCTTAGTTACTCCCCCACTGTTTG
YFP^N-COP1-F	ACGCGTCGACAATGGAAGAGATTTCGACGG
YFP^N-COP1-R	GACTAGTTCACGCAGCGAGTACCAGAAC
YFP^C-GH3.5-F	GGACTAGTATGCCTGAGGCACCAAAG
YFP^C-GH3.5-R	CGGGATCCTTAGTTACTCCCCCACTG
His-UBC10-F	CAAATGGGTCGCGGATCCATGGCGTCGAAGCGGATCTTG
His-UBC10-R	TCGACGGAGCTCGAATTCTTAGCCCATGGCATACTTCTG
738-MYC-F2	AACGATAGCCATGGAGTCGACGATGGAACAAAAGCTAATC
MYC-Ub-MF	TCAGAAGAGGATCTAATGCAGATCTTTGTT
MYC-Ub-MR	AACAAAGATCTGCATTAGATCCTCTTCTGA
738-Ub-R2	CATCGTATGGGTAAAGCGGCCGCGTTTACTAACCACCACGGAGCC
GH3.5-F1	TTTTCTGATTAACAGGAATTCATGCCTGAGGCACCAAAG

GH3.5-MF	CAAACAGTGGGGGAGTAACATGGTGAGCAAGGGCGAG
GH3.5-MR	CTCGCCCTTGCTCACCATGTTACTCCCCCACTGTTTG
GFP-R1	GTCGACTCTAGAGGATCCTTACTTGTACAGCTCGTC
**For generating *cop1-4l1* and *cop1-4l3 gh3-septuple* mutants by CRISPR**	
cop1-4l-sgRNA1-F	ATATATGGTCTCGATTGAATCAGTCAACTGTCTCAAGTT
cop1-4l-sgRNA1-R	TGAATCAGTCAACTGTCTCAAGTTTTAGAGCTAGAAATAGC
cop1-4l-sgRNA2-F	AACCCTTGTCTACTCAACATCTCAATCTCTTAGTCGACTCTAC
cop1-4l-sgRNA2-R	ATTATTGGTCTCGAAACCCTTGTCTACTCAACATCTC
**For genotyping**	
cop1-4-seqF	GGCCACATGAGAAGAACCAGATT
cop1-4-seqR	CACAGATTGAAAATCTGCAAGGC
LBb1.3	ATTTTGCCGATTTCGGAAC
gh3.5-1-LP	GAGATGGGTTTGGAAGAGAGG
gh3.5-1-RP	AGTATGGAGGAATGATTTGCG
gh3.6-LP	AAACCTAAACGATGCCTGAGG
gh3.6-RP	CTCAGGCCAATGTTTCTCAAG
gh3.5-GT-F	ATCCGGTACAGGTTTTGACCA
gh3.5-GT-R	TTCTTTGTGGATGCGTCATTCT
gh3.6-GT-F	GCTCCCCGAATTCAAGATATGC
gh3.6-GT-R	GGATGATTCTTGTTCAACTCTCGG
gh3.9-GT-F	GTGGCCCCCTTGTTGAGATT
gh3.9-GT-R	CGCATCCAGGGAATCGAACC
gh3.17-GT-F	GAGTATAGTGAAGACCAAGTGAGT
gh3.17-GT-R	TGTCCCTGTTCCTGCATCTC
**For RT-qPCR**	
GH3.5-qF	CCGGGCTTTGCAGATACAGA
GH3.5-qR	CTCGTCGGTCTTGTCGGAAT
PP2A-qF	TTCGTATCGGTGGTTCTTCTCC
PP2A-qR	ACGAACTTTCAGTGCTACCAA

Supplementary Table 2. Parameters for UPLC-MS/MS analysis

For analysis of in vitro IAA-Asp conjugates

UPLC parameters

Time (min)	Solvent Ratio B(%)	Flow (m/min)	A	B	Column temp (°C)	Retention time (min) (IAA-Asp)
0	5	0.3	water containing 0.1% formic acid	Meth	30	2.84
0.3	5					
1	41					
4	45					
4.2	80					
4.5	100					
6.5	100					
6.7	5					
8	5					

MS parameters

ESI	Spray voltage (V)	Resolution	Capillary temp (°C)	Scan Begin (m/z)	Scan end (m/z)	Sheath gas flow (Arb)	Auxiliary gas flow (Arb)	Aux gas heater temp (°C)
Positive	3800	70000	320	100	350	40	11	370

For analysis of endogenous IAA and IAA metabolites

UPLC parameters

Time (min)	Solvent Ratio B(%)	Column temp (°C)
0	5	40
8	40	
9	90	
12	90	
12.5	5	
15	5	

MS parameters

Name	ESI	Q1	Q3
IAA	Positive	176.1	130
oxIAA	Positive	192.1	146
IAA-Asp	Positive	291.2	103
oxIAA-Asp	Positive	307.2	146
IAA-Glu	Positive	305.2	103
oxIAA-Glu	Positive	321.1	146
Trp	Positive	205.1	118
IPyA	Positive	204.1	144
