# Supplementary material for: Inactivation of GH3.5 by COP1-mediated K63-linked ubiquitination promotes seedling hypocotyl elongation
Source: Nat Commun. 2025 Apr 14;16:3541. doi: 10.1038/s41467-025-58767-6 (PMC11997217; doi:10.1038/s41467-025-58767-6)
Supplement: Supplementary file 2 — Reporting Summary [file 41467_2025_58767_MOESM2_ESM.pdf]

nature portfolio

Corresponding author(s): Jian Li

Last updated by author(s): Feb 28, 2025

Reporting Summary

Nature Portfolio wishes to improve the reproducibility of the work that we publish. This form provides structure for consistency and transparency in reporting. For further information on Nature Portfolio policies, see our Editorial Policies and the Editorial Policy Checklist.

Statistics

For all statistical analyses, confirm that the following items are present in the figure legend, table legend, main text, or Methods section.

n/a	Confirmed	
☐	☒	The exact sample size (*n*) for each experimental group/condition, given as a discrete number and unit of measurement
☐	☒	A statement on whether measurements were taken from distinct samples or whether the same sample was measured repeatedly
☐	☒	The statistical test(s) used AND whether they are one- or two-sided<br>*Only common tests should be described solely by name; describe more complex techniques in the Methods section.*
☒	☐	A description of all covariates tested
☒	☐	A description of any assumptions or corrections, such as tests of normality and adjustment for multiple comparisons
☐	☒	A full description of the statistical parameters including central tendency (e.g. means) or other basic estimates (e.g. regression coefficient) AND variation (e.g. standard deviation) or associated estimates of uncertainty (e.g. confidence intervals)
☐	☒	For null hypothesis testing, the test statistic (e.g. *F*, *t*, *r*) with confidence intervals, effect sizes, degrees of freedom and *P* value noted<br>*Give P values as exact values whenever suitable.*
☒	☐	For Bayesian analysis, information on the choice of priors and Markov chain Monte Carlo settings
☒	☐	For hierarchical and complex designs, identification of the appropriate level for tests and full reporting of outcomes
☒	☐	Estimates of effect sizes (e.g. Cohen's *d*, Pearson's *r*), indicating how they were calculated

Our web collection on statistics for biologists contains articles on many of the points above.

Software and code

Policy information about availability of computer code

Data collection	Detection of gene expression was performed with the Real-time PCR Cycler (ABI QuantStudio 6 Flex Real-Time PCR System, Thermo Fisher Scientific); the chemiluminescent imaging system (Tanon 5200) was used for Western blot; Night SHADE LB 985 (Berthold Technologies) was used for detection of luciferase signals; The Image J software (https://imagej.nih.gov/ij/) (version 1.52a) was used to measure the hypocotyl length of Arabidopsis seedlings. The Zeiss LSM880 confocal microscope was used to monitor the fluorescence signals from the BiFC assay and GH3.5-GFP seedlings. The production of IAA-Asp in vitro was measured by UPLC–MS/MS (Thermo Vanquish UHPLC system; Q Exactive mass spectrometer; Thermo Scientific, USA). Raw data were analyzed using Xcalibur 2.1 (Thermo Scientific, USA) with default settings. The peak of IAA-Asp was detected in the mass range (m/z 291.0949-291.0978). The measurement of IAA and IAA metabolites in vivo was performed by UHPLC-MS/MS (Thermo Scientific Ultimate 3000 UHPLC coupled with TSQ Quantiva). Data acquisition and analysis was performed using a Thermo Scientific Xcalibur 2.1 data system.
Data analysis	Microsoft Office Excel software (version 2007) was used to analyze qRT-PCR results; GraphPad Prism software (version 8.0.2) was used to determine statistical significance based on a two-tailed Student's t-test, one-way or two-way ANOVA with Tukey's post hoc test; The targets of CRISPR/Cas9 were selected using the web tool CRISPR-P(version 2.0, http://crispr.hzau.edu.cn/CRISPR/).

For manuscripts utilizing custom algorithms or software that are central to the research but not yet described in published literature, software must be made available to editors and reviewers. We strongly encourage code deposition in a community repository (e.g. GitHub). See the Nature Portfolio guidelines for submitting code & software for further information.

Data

Policy information about availability of data

All manuscripts must include a data availability statement. This statement should provide the following information, where applicable:

- Accession codes, unique identifiers, or web links for publicly available datasets
- A description of any restrictions on data availability
- For clinical datasets or third party data, please ensure that the statement adheres to our policy

All data supporting the findings of this study are available within the paper and its Supplementary Information. Source data are provided with this paper.

Research involving human participants, their data, or biological material

Policy information about studies with human participants or human data. See also policy information about sex, gender (identity/presentation), and sexual orientation and race, ethnicity and racism.

Reporting on sex and gender	n/a
Reporting on race, ethnicity, or other socially relevant groupings	n/a
Population characteristics	n/a
Recruitment	n/a
Ethics oversight	n/a

Note that full information on the approval of the study protocol must also be provided in the manuscript.

Field-specific reporting

Please select the one below that is the best fit for your research. If you are not sure, read the appropriate sections before making your selection.

☒ Life sciences ☐ Behavioural & social sciences ☐ Ecological, evolutionary & environmental sciences

For a reference copy of the document with all sections, see nature.com/documents/nr-reporting-summary-flat.pdf

Life sciences study design

All studies must disclose on these points even when the disclosure is negative.

Sample size	Approximately 30 4-day-old seedlings for each sample were used for the measurement of hypocotyl length. No statistical method was used to predetermine sample sizes.
Data exclusions	No data were excluded from analyses.
Replication	Experiments were performed three times with similar results.
Randomization	All samples were arranged randomly into experimental groups.
Blinding	Animal experiments were not performed in this study, thus the investigators were not blinded to the experiments reported in this study.

Reporting for specific materials, systems and methods

We require information from authors about some types of materials, experimental systems and methods used in many studies. Here, indicate whether each material, system or method listed is relevant to your study. If you are not sure if a list item applies to your research, read the appropriate section before selecting a response.

Materials & experimental systems

n/a	Involved in the study
☐	☒ Antibodies
☒	☐ Eukaryotic cell lines
☒	☐ Palaeontology and archaeology
☒	☐ Animals and other organisms
☒	☐ Clinical data
☒	☐ Dual use research of concern
☐	☒ Plants

Methods

n/a	Involved in the study
☒	☐ ChIP-seq
☒	☐ Flow cytometry
☒	☐ MRI-based neuroimaging

Antibodies

Antibodies used

Anti-COP1 is custom-made antibody. Other antibodies used in this study are commercially available, including anti-MBP (#E8032S, New England Biolabs), anti-GST (AE006, Abclonal), anti-Flag (F3165-.2MG, Sigma-Aldrich), anti-MYC (C3956, Sigma-Aldrich), anti-GFP (M20004, Abmart), anti-Actin (AC009, Abclonal), anti-Histone H3 (H0164, Sigma-Aldrich), anti-HSP (AbM51099-31-PU, Beijing Protein Innovation), anti-pan-Ub (sc-8017, Santa Cruz), anti-K63-Ub (5621S, Cell Signaling Technology), and anti-K48-Ub (8081S, Cell Signaling Technology).

Validation

The anti-COP1 antibody was previously validated (McNellis et al., 1994, Plant Cell; Zhou et al., 2024, Nature Communications). The validation of anti-MBP (1:5000 dilution) can be found on the website: https://www.neb.cn/zh-cn/products/e8032-anti-mbp-monoclonal-antibody. The validation of anti-GST (1:1000 dilution) can be found on the website: https://abclonal.com.cn/catalog/AE006. The validation of anti-Flag (1:2000 dilution) can be found on the website: https://www.sigmaaldrich.cn/CN/zh/product/sigma/f3165. The validation of anti-MYC (1:2000 dilution) can be found on the website: https://www.sigmaaldrich.cn/CN/zh/product/sigma/c3956. The validation of anti-GFP (1:5000 dilution) can be found on the website: https://www.ab-mart.com.cn/page.aspx?node=%2059%20&id=%20971. The validation of anti-Actin (1:2000 dilution) can be found on the website: https://abclonal.com.cn/catalog/AC009. The validation of anti-Histone H3 (1:5000 dilution) can be found on the website: https://www.sigmaaldrich.cn/CN/zh/product/sigma/h0164. The validation of anti-HSP (1:5000 dilution) can be found on the website: http://www.proteomics.org.cn/product/202.html. The validation of anti-pan-Ub (1:1000 dilution) can be found on the website: https://www.scbt.com/zh/p/ubiquitin-antibody-p4d1. The validation of anti-K63-Ub (1:1000 dilution) can be found on the website: https://www.cellsignal.com/products/primary-antibodies/k63-linkage-specific-polyubiquitin-d7a11-rabbit-mab/5621. The validation of anti-K48-Ub (1:1000 dilution) can be found on the website: https://www.cellsignal.com/products/primary-antibodies/k48-linkage-specific-polyubiquitin-d9d5-rabbit-mab/8081.

Dual use research of concern

Policy information about dual use research of concern

Hazards

Could the accidental, deliberate or reckless misuse of agents or technologies generated in the work, or the application of information presented in the manuscript, pose a threat to:

No	Yes
☒	☐ Public health
☒	☐ National security
☒	☐ Crops and/or livestock
☒	☐ Ecosystems
☒	☐ Any other significant area

Experiments of concern

Does the work involve any of these experiments of concern:

No	Yes	
☒	☐	Demonstrate how to render a vaccine ineffective
☒	☐	Confer resistance to therapeutically useful antibiotics or antiviral agents
☒	☐	Enhance the virulence of a pathogen or render a nonpathogen virulent
☒	☐	Increase transmissibility of a pathogen
☒	☐	Alter the host range of a pathogen
☒	☐	Enable evasion of diagnostic/detection modalities
☒	☐	Enable the weaponization of a biological agent or toxin
☒	☐	Any other potentially harmful combination of experiments and agents

Plants

Seed stocks	The Arabidopsis Columbia (Col) ecotype was from our own laborotary. The gh3.5-1 (SALK_033434C, Pierdonati E et al., 2019, Plants), gh3.6 (SALK_013458C, González-Lamothe R et al., 2012, Plant Cell), gh3(5/6/9/17) (Guo et al., 2022, Biochem Biophys Res Commun.), gh3(1/2/3/4/5/6/17) (Guo et al., 2022, Biochem Biophys Res Commun.), ubc35-1 ubc36-1 (Wen et al., 2014 Plant J; Romero-Barrios et al., 2020 Plant Cell), cop1-4 (McNellis et al., 1994, Plant Cell), cop1-6 (McNellis et al., 1994, Plant Cell), and YFP-COP1/cop1-6 (Lin et al., 2017, PNAS) were previously described.
Novel plant genotypes	UBQpro:GH3.5-GFP was generated by floral dip method, T3 generation and three independent lines were analyzed. The gh3.5-1 gh3.6, cop1-4 gh3 and UBQpro:GH3.5-GFP/cop1-4 were generated by genetic crossing. cop1-4l1 and cop1-4l3 gh3-septuple mutants were generated by CRISPR/Cas9, sgRNA sequence were provided in supplementary Table 1.
Authentication	Genotype of gh3.5-1, gh3.6 and ubc35-1 ubc36-1 mutants were identified using a PCR-based method. gh3(5/6/9/17), gh3(1/2/3/4/5/6/17), cop1-4 and cop1-6 mutants were determined by sequencing. YFP-COP1/cop1-6 transgenic plants were determined by western blot.
